# Stroke Recovery: Surprising Influences and Residual Consequences

**DOI:** 10.1155/2014/378263

**Published:** 2014-11-11

**Authors:** Argye E. Hillis, Donna C. Tippett

**Affiliations:** ^1^Department of Neurology, Johns Hopkins University School of Medicine, Baltimore, MD 21287, USA; ^2^Department of Physical Medicine and Rehabilitation, Johns Hopkins University School of Medicine, Baltimore, MD 21287, USA; ^3^Department of Cognitive Science, Krieger School of Arts and Sciences, Johns Hopkins University, Baltimore, MD 21218, USA; ^4^Department of Otolaryngology-Head and Neck Surgery, Johns Hopkins University School of Medicine, Baltimore, MD 21287, USA

## Abstract

There is startling individual variability in the degree to which people recover from stroke and the duration of time over which recovery of some symptoms occurs. There are a variety of mechanisms of recovery from stroke which take place at distinct time points after stroke and are influenced by different variables. We review recent studies from our laboratory that unveil some surprising findings, such as the role of education in chronic recovery. We also report data showing that the consequences that most plague survivors of stroke and their caregivers are loss of high level cortical functions, such as empathy or written language. These results have implications for rehabilitation and management of stroke.

## 1. Introduction

Stroke is among the leading causes of serious, long-term disability worldwide; 15 million people suffer a stroke each year. Almost six million people die of stroke annually, and another five million people have permanent disability due to stroke (http://www.world-heart-federation.org/cardiovascular-health/stroke). Yet, physicians are notoriously weak in predicting who will recover from stroke, how much they will recover, and when they will recover. It is widely recognized that there is a great deal of individual variability in stroke recovery. Even two individuals with very similar appearing ischemic strokes may show very different outcomes one year later. In this paper, we review recent studies from our research group, the Stroke Cognitive Outcomes and Recovery (SCORE) Lab, revealing new insights into sequelae of stroke that are most important to survivors and caregivers and the variables that influence cognitive recovery after stroke. These data have implications for both acute management of stroke and the need to explore new avenues of rehabilitation.

### 1.1. Why Focus on Cognitive Recovery?

The human brain is responsible for all of the functions that define who we are and how we relate to one another—our talents, our intellect, our creativity, our ability to participate in sports, our ability to communicate, and our ability to understand and share in the emotions of others. Stroke can interfere with any or all of these functions. Most of the brain, in fact, supports cognitive and integrative processes underlying complex systems, such as attention, working memory, cognitive control, and language that are critical for these activities. Yet, stroke outcomes research traditionally has focused on recovery of very basic activities of daily living, such as feeding oneself and walking. Consider the most commonly used outcome measures for stroke intervention trials, the Modified Rankin Scale (MRS) [1], the Barthel Index (BI) [2], and the National Institutes of Health Stroke Scale (NIHSS; http://www.ninds.nih.gov/doctors/nih_stroke_scale.pdf). An MRS score of 3 corresponds to moderate disability, defined as “requires some help, but able to walk without assistance.” An MRS score of 2, slight disability, is defined as “unable to carry out all previous activities, but able look after own affairs without assistance.” A score of 2 encompasses the status of all of those individuals who are unable to go back to their previous work because of mild or moderate language or cognitive deficits (e.g., affecting spelling, grammar, and executive function), loss of creativity, impaired emotional regulation, or loss of empathy that interferes with interpersonal relationships, as long as these deficits are not severe enough to interfere with looking after one's own affairs. Artists, executives, physicians, lawyers, and so on might be disabled from returning to previous vocations by deficits that would not interfere with some other vocations. Thus, a given higher level cortical deficit might yield an MRS score of 2 in one person and a score of 1 (no significant disability despite symptoms) in another. Furthermore, an individual might show substantial, meaningful recovery in higher cortical function over time without showing any change in the MRS scale. Likewise, the BI captures only the status of feeding, bathing, grooming, dressing, bowel function, bladder function, toilet use, transfers, mobility on level surfaces, and mobility on stairs. While this scale may measure how easy or difficult it is to care for someone after a large stroke, many people recover completely in all of these functions but remain unable to return to work or previous social roles because of residual deficits in higher cortical functions. Moreover, recovery of these basic functions is not what makes stroke survivors or their caregivers happy. A recent longitudinal study of 399 stroke survivors and their caregivers found that caregivers reported greater sense of well-being when the stroke survivor had more severe stroke, but fewer symptoms of depression and better cognitive function [3]. Individuals with cognitive impairment after stroke have poorer functional recovery, higher rates of depression, and even higher mortality after stroke [4, 5].

The NIHSS also has only a few items that evaluate cognitive function (particularly right hemisphere cognitive functions), but many more points that evaluate motor function (e.g., 8 points for holding up the arm and the leg on one side). This limitation has important consequences for evaluating both candidates for treatment and outcomes of treatment. For example, several studies have shown that the NIHSS score underestimates the volume of ischemia in patients with right hemisphere stroke relative to left hemisphere stroke [6–8]. Because of this limitation, the NIHSS may underestimate response to reperfusion of the cortex, particularly after right hemisphere stroke, as illustrated in Section 2.1.1 [9]. To address this limitation, Gottesman and colleagues [10] evaluated whether adding greater weight to right hemisphere cortical dysfunction (hemispatial neglect and extinction) would improve its correlation with volume of infarct. In a study of 200 individuals with acute stroke with concurrent NIHSS, cognitive testing, and MRI, they showed that adding a few simple quantitative tests of neglect and extinction to the NIHSS improved its detection of right and left hemisphere ischemia and its correlation with volume of infarct.

Thus, it is possible that some of the treatments that have failed to show benefit in acute stroke trials have “failed” simply because they have not measured changes in cognitive function. Often, in large vessel stroke, there is early infarct in the deep subcortical areas (e.g., lenticulostriate territory) with surrounding hypoperfused cortex that may be salvageable. The motor deficits due to deep infarct may not recover. But if there is reperfusion of the cortex, cognitive function may be restored (as discussed later in Section 2.1.2). Therefore, it is crucial to include adequate evaluation of cortical function to measure the effects of acute intervention. Recent clinical trials in stroke are just beginning to include cognitive endpoints (e.g., [11]), but trials aiming at reperfusion typically have not included such endpoints.

### 1.2. Surprising Sequelae of That Stymie Stroke Survivors

Previous studies that have investigated quality of life (QOL) or health related quality of life QOL (HRQOL) after stroke have focused on motor function, communication, and activities of daily living, using instruments that survey participants about stroke sequelae which typically are evaluated by medical personnel [12, 13]. Studies have found that age, nonwhite race, impaired upper-extremity function, and greater number of comorbidities are all associated with reduced HRQOL within the physical domain. A larger number of comorbidities are also associated with poorer HRQOL in the domain of memory and thinking, and stroke survivors whose hemiparesis affected the dominant side or had ischemic (rather than hemorrhagic) stroke reported poorer HRQOL in the domain of communication (QOL) [12]. Several studies have shown that depression is strongly correlated with QOL measured with traditional HRQOL instruments for stroke [14, 15].

We carried out a pilot study to identify the sequelae that were most important to stroke survivors and caregivers. This study was motivated by the observation that individuals who were recovering from stroke sometimes assigned surprisingly different values to various consequences of stroke, compared to values assigned by their family members or professionals. An additional motivation was the observation that stroke survivors or their caregivers frequently reported problems that are not typically measured by stroke scales—difficulty in sleep or sex, overwhelming fatigue, change in personality, and so on. As it is critical to understand what sequelae have the greatest impact on QOL of survivors and their caregivers to focus poststroke interventions, we created new questionnaires, including questions about all the sequelae noted above, as a preliminary investigation of the impact of various consequences of stroke. The appendix includes a list of these items.

We surveyed 33 stroke patients and 28 caregivers of the same stroke patients in our Stroke Prevention and Recovery Center (SPARC) using questionnaires about possible stroke sequelae [16]. They were asked to rate residual problems in two ways: (1) from most to least important in terms of the impact on QOL and (2) as severe, moderate, mild, or not a problem. Symptoms included change in personality or behavior, motor function (weakness, clumsiness, etc.), motor speech, word retrieval, reading, writing, memory, attention, spatial perception (neglect of one side), other cognitive problems, sensation (vision, numbness/tingling, pain, etc.), mood, walking, swallowing, sleep, empathy (understanding emotions of others and expressing emotion through tone of voice and facial expression), pain, fatigue, and sexual function (see the appendix).

Stroke survivors were on average 66 (31–83) years old and were surveyed at an average of 22.2 months after stroke; 42% were women. Diagnoses included 14 left hemisphere, 14 right hemisphere, 3 bilateral, and 2 brainstem strokes. We identified symptoms that were rated as the top 5 most important residual problems and/or at least “moderate” problems. The single most frequently reported important/moderate consequence by both survivors of left hemisphere stroke and their caregivers was difficulty in spelling and/or writing (identified by 71% of each) (see Table 1). Word-retrieval and mood problems were also frequently reported (by 57% of caregivers), as was right-sided weakness (by 57% of survivors). Right hemisphere stroke survivors themselves reported few residual deficits, but equally common were: fatigue, left-sided weakness, problems with mood, reading, writing, memory, and sexual function (with symptoms in each of these domains rated as important/moderate problem by 21% of right hemisphere stroke survivors). The most frequently reported important/moderate consequence by caregivers of right hemisphere stroke survivors was impaired recognition of the emotions of others (loss of emotional empathy), identified by 50% of caregivers, followed by “other cognitive problems,” “change in personality and behavior,” and “walking” (Table 1).

These results reveal that deficits in spelling/writing after left hemisphere stroke and loss of empathy after right hemisphere stroke are probably underestimated as residual consequences of stroke. Spelling has taken on new importance in a community that relies on email, texting, and online shopping and banking. The importance of empathy in communication and social relationship has been understood by social scientists for decades, but little attention has been given to impairments of empathy after stroke [17]. Efforts to understand the variables that mediate these deficits and interventions to alleviate these problems are essential to improve QOL after stroke.

## 2. Mechanisms of Stroke Recovery

In general, the deficits caused by stroke are the most severe at onset and gradually improve over time although the most rapid recovery (especially in motor function) often occurs in the first three months [18]. In a large study of chronic aphasia recovery described below, Hope and colleagues [19] found that the single most important determinant of recovery of speech production was time since onset of stroke, indicating that improvement continues over time, even in the chronic stage. The brain recovers from a focal lesion like stroke through a variety of mechanisms that take place at different times after onset [20, 21]. Here we briefly review some of these mechanisms, focusing on restoring blood flow to critical brain regions and reorganization of structure-function relationships.

### 2.1. Early Cognitive Recovery Depends on Degree and Location of Reperfusion

#### 2.1.1. Restoring Blood Flow Improves Cognitive Function, Even When There Is No Change in NIHSS

The focus of acute stroke interventions, such as thrombolysis, embolectomy, stenting, and transcranial Doppler ultrasound-augmented clot disruption, is to restore blood flow to ischemic tissue that is receiving enough blood to survive, but not enough to function (the so-called “ischemic penumbra”). In most cases, the ischemic tissue that is salvageable is largely limited to the cortex. Yet, most acute stroke trials have measured response to treatment using scales that are insensitive to change in cortical function, such as the MRS and BI, or are heavily weighted toward assessment of motor function, such as the NIHSS as described earlier.

In an initial investigation, we studied 10 patients with acute, nondominant hemisphere stroke who were candidates for intervention to restore perfusion, based on having a small acute stroke (measured on diffusion-weighted imaging or DWI), but a larger area of hypoperfusion (measured on perfusion-weighted imaging or PWI), and a visualized clot or area of stenosis in cerebral vessel. They underwent DWI, PWI, NIHSS, and a simple line cancellation test (a test of hemispatial neglect) on Days 1 and 3. We calculated correlations between change in volume of stroke, change in perfusion abnormality (defined as time to peak delay of contrast in a region of interest relative to the homologous region of interest in the opposite hemisphere), and change in functional tests. Initial NIHSS score ranged from 1 to 16 (mean = 9). Initial score on the line cancellation test was ranged from 12% to 93% (mean = 55.5%) errors. Volume of infarct on DWI ranged from 3 to 31 cm^3^ (mean = 8.9 cm^3^). Volume of PWI abnormality ranged from 55 to 284 cm^3^ (mean = 156 cm^3^). Notably, all of these patients had large areas of hypoperfusion beyond the infarct and were considered candidates for intervention to restore blood flow. Intervention included endovascular treatment, urgent endarterectomy, and temporarily induced blood pressure elevation [22]. With intervention, change in NIHSS score ranged from −5 to 0 (mean = −1.7). Change in line cancellation ranged from −39.6 to +14.6 (mean = −14.3 cm^3^). Change in infarct volume ranged from −4 to 32 cm^3^ (mean = 4.3 cm^3^). Change in PWI abnormality ranged from −209 to 0 cm^3^ (mean = −70.2 cm^3^). Change in volume of hypoperfused tissue on PWI correlated with change in line cancellation performance (*r* = 0.83; *P* = 0.003) but did not correlate with change in NIHSS score (*r* = 0.26; *P* = NS). This study provided evidence that improvement in perfusion was associated with improvement in a simple measure of cognitive function, even when it was not associated with improvement in the NIHSS score [9].

#### 2.1.2. Restoring Blood Flow to Specific Areas Results in Early Recovery of Specific Cognitive Function

In a series of studies, we tested the hypothesis that improvement in cortical function depends not only on how much tissue is reperfused but also on the location of the cortex that is reperfused.

**Figure 1:**
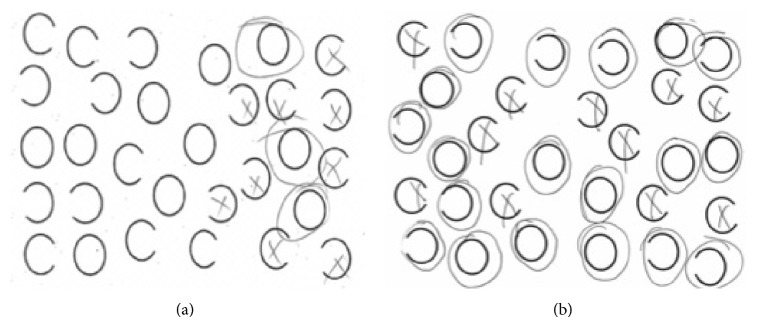
Contrasting performance of patients with viewer-centered and stimulus-centered neglect on the gap-detection test [24] (in this task the patient is asked to circle all the complete circles and put an X over circles that have a gap on the left or right side). (a) Performance by a patient with a right frontoparietal stroke and viewer-centered neglect on the gap-detection test. Note that he misses all of the stimuli on the left side of the view but detects left gaps in circles on the right view. (b) Performance by a patient with a right temporal stroke and stimulus-centered neglect on the gap-detection test. Note that he fails to detect the left gaps in circles on the right and left sides of the view.

**Figure 2:**
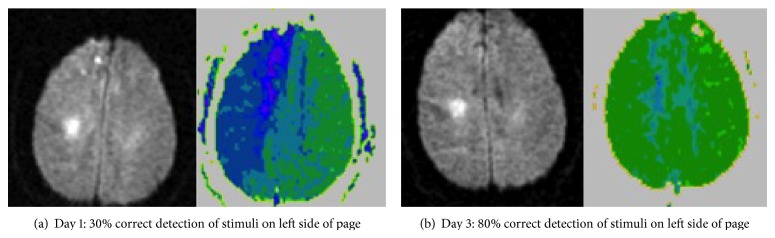
(a) Diffusion-weighted image (DWI; left), showing small subcortical infarct and perfusion-weighted image (PWI; right) of a patient with severe viewer-centered neglect at Day 1. (b) DWI and PWI of the same patient at Day 3, after viewer-centered neglect recovered, as indicated by recovery of detecting stimuli on the left and copying stimuli on the left of a scene. PWI shows that reperfusion of the right frontoparietal cortex was associated with recovery of viewer-centered neglect. In this case, reperfusion was brought about with induced blood pressure elevation.

**Figure 3:**
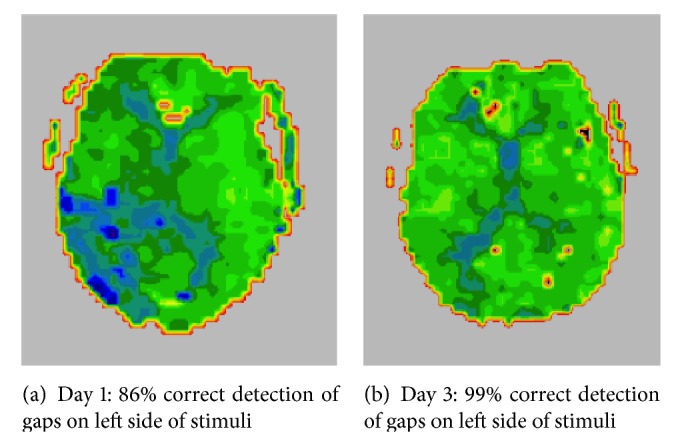
(a) PWI of a patient with severe stimulus-centered neglect at Day 1. (b) PWI of the same patient at Day 6, after stimulus-centered neglect recovered, as indicated by recovery of detecting gaps on the left sides of circles (irrespective of their location w.r.t. the viewer) and copying the left halves of stimuli on both sides of a scene. PWI shows that reperfusion of the right temporal cortex was associated with recovery of stimulus-centered neglect. In this case, reperfusion was brought about by urgent carotid endarterectomy.

In one recent study, we evaluated the hypothesis that restoring blood flow to specific cortical regions in the right hemisphere after acute stroke results in improvement in distinct variants of hemispatial neglect (viewer-centered neglect versus stimulus-centered neglect) [23]. These two forms of neglect are shown in Figure 1. Previous studies have shown that these two forms of neglect result from different locations of stroke [24–26]. Twenty-five patients with acute right stroke were evaluated at Day 1 and Days 3–5 with a battery of neglect tests and diffusion- and perfusion-weighted MR Imaging. We used multivariate linear regression analysis to identify areas where reperfusion predicted degree of improvement in scores on each type of neglect, independently of reperfusion of other areas, total change in the volume of infarct or hypoperfusion (defined as >4 second delay in time to peak arrival of contrast, relative to homologous voxels on the left), and age. The stroke patients were on average 65.5 (± SD 16.1) years old. At onset, 8 (30%) had viewer-centered neglect; 6 (22%) had stimulus-centered neglect plus viewer-centered neglect. Mean infarct DWI at onset was 23.1 (±27.2) cc. The mean volume of hypoperfusion on PWI was 94.6 (±85.5) cc. They received a variety of interventions to restore blood flow, including carotid stenting, urgent endarterectomy, endovascular therapy, thrombolysis, and temporarily induced blood pressure elevation. The mean change in volume of ischemia on DWI was 3.2 (±18.5) cc increase (growth in infarct); the mean change in volume of hypoperfusion on PWI was −35.1 (±55.0) cc or improvement in perfusion. Multivariate linear regression analysis revealed specific Brodmann areas (BA) where reperfusion was associated with improvement in viewer- or stimulus-centered neglect, independently of reperfusion of other regions and independently of age and change in volume of infarct and hypoperfusion. Analyses revealed that reperfusion of dorsal frontoparietal cortex (right BA 46, 4, 40) independently predicted improvement in viewer-centered neglect, such as detecting stimuli on the left side of the page and copying left stimuli in the scene (*r* = 0.951; *P* < 0.0001), as illustrated in Figure 2. Reperfusion of right temporooccipital cortex (right BA 37, 18, 38) independently contributed to improvement in stimulus-centered neglect, measured by detecting left gaps in circles on both sides of the page (*r* = 0.926; *P* < 0.0001), as illustrated in Figure 3. These results confirmed that restoring blood flow to specific cortical regions yields improvement in different types of neglect [23].

Likewise, in several additional studies, we tested the hypothesis that reperfusion of the distinct cortical regions of the left hemisphere, in the absence of infarct in that region, would restore the associated language function [27]. In one study, we investigated five patients with impaired word meaning associated with poor perfusion, but not infarction, in superior temporal cortex, and one patient with a superimposed deficit in word retrieval, associated with poor perfusion of left inferior temporal cortex. Each patient was treated to increase perfusion of the ischemic and dysfunctional tissue. Daily testing of naming and comprehension, with stimuli matched for difficulty, showed improvement in word meaning in the patients who showed reperfusion of left superior temporal cortex and showed improvement in oral naming (but not word meaning) in the patient who showed reperfusion of left inferior temporal cortex [27]. In another study, reperfusion of inferior temporal cortex (within BA 37) was the area most strongly associated with improvement in naming in patients with acute left hemisphere stroke [28]. Yet another study showed that reperfusion of left inferior frontal cortex was associated with improvement specific to writing verbs [29]. These results illustrate that reperfusion of specific brain regions results in recovery of distinct language functions.

## 3. Surprising Patient Variables That Influence Cognitive Recovery

Several studies have demonstrated that motor or language recovery is significantly associated with volume of infarct, although the association is relatively weak [30]. A great deal of variance in recovery of cognitive functions remains unexplained, even after accounting for lesion volume. For example, in a study of 270 (mostly left hemisphere) stroke patients, recovery of speech production (a composite score) correlated with volume of infarct (*r* ^2^ = 0.35, *F* = 144.73, *P* < 0.001) [19]. In that study, the correlation improved when information about site of lesion was added. Recovery of speech production was best predicted by subset of 37 variables (*r* ^2^ = 0.59, *F* = 38.38, *P* < 0.001), including time after stroke (which was the most significant, single predictor), volume of stroke, and involvement of 35 different brain regions. Recovery of speech production was not predicted by a combination of time since stroke, age at stroke, premorbid handedness, and gender. The role of education was not evaluated in that study. We hypothesized that education might have a role in recovery from cognitive sequelae of stroke, based on previous studies indicating that education may promote neuroplasticity or may have a neuroprotective effect against cognitive decline [31]. The proposal that education provides “cognitive reserve” that reduces the risk of dementia has received support from a variety of sources [32–35]. That is, higher education may provide more general cognitive resources on which to rely and thus delay the onset of dementia. However, the role of education in recovery from stroke has been less well studied. One study did find that the highest educational levels were associated with lower rates of poststroke cognitive deficits and dementia and higher rates of long-term survival, independently of stroke severity, age, sex, marital status, and white matter lesions in individuals with mild/moderate ischemic stroke [36]. Results were interpreted as support for the hypothesis that high education, a proxy for cognitive reserve, protects against poststroke cognitive impairment.

### 3.1. The Effects of Education and Antidepressant Use on Language Recovery

We tested the hypothesis that degree of recovery beyond 3 months is influenced not only by lesion volume but also independently by education. We tested 45 acute left hemisphere ischemic stroke patients. Their mean age was 54.9 years; range was 18 to 90 years; mean education was 14.7 years; range was 6 to 20 years. They were studied on average for 35.4 months after onset of stroke on the Western Aphasia Battery (WAB). The primary outcome variable was a summary score of comprehension, repetition, and naming summary scores from the WAB. “Spontaneous speech” fluency and content scores were omitted, because these scores are subjective rating scores and have a lower interrater reliability in scoring than the objective scores on the other subtests. Infarct volume was measured on follow-up MRI obtained at the time of testing. We determined variables associated with WAB Quartile (because scores were not normally distributed) using multivariable logistic regression, with antidepressant use (from onset of stroke through recovery) as cofactor, and age, education, infarct volume, and time postonset (TPO) as covariates. Individuals who were prescribed antidepressants at onset consistently stayed on the medication, although doses were adjusted. Nearly all antidepressants were selective serotonin reuptake inhibitors (SSRIs); a small percentage consisted of venlafaxine (which has SSRI and tricyclic properties) or tricyclic antidepressants.

We found that final WAB Quartile was significantly predicted by a model that included education, age, volume of infarct, and antidepressant use but did not include time since onset (chi-square for goodness of fit = 207; *P* < 0.0001; Cox and Snell *r* ^2^ = 0.47). Education had the highest Wald statistic of 17.3 (*P* < 0.0001) [df = 1], followed by volume of infarct (Wald = 7.0; *P* = 0.008), antidepressant use (Wald = 5.2; *P* = 0.022), and age (Wald = 5.0; *P* = 0.023). Compared to individuals who had never taken antidepressants, those taking antidepressants had higher repetition scores (mean 9.4 versus 7.6; *P* = 0.039), even though they had larger infarct (mean 225 versus 82 cc; *P* = 0.008); they were no differences in age, education, TPO, or total WAB (mean 28.4 versus 22.9). These results show that better chronic aphasia recovery is associated with higher education and current antidepressant use, as well as smaller lesion size and younger age (independently of one another). Although our study was smaller than the study by Hope and colleagues [19], the effects of education and antidepressant use were so powerful, that, even with lower power, they had a highly significant effect independently of lesion size [37]. The positive effect of antidepressant use (mostly SSRIs) is consistent with a recent clinical trial showing positive effects of fluoxetine on motor recovery after acute stroke, independently of the effects on depression [38], as well as a recent study of the effects of SSRIs on dependence, overall neurological impairment, depression, and anxiety after stroke [39].

The effect of education on very simple language tests is somewhat surprising. The WAB tests can be performed easily by healthy individuals with a grade school education; they include tasks of naming familiar objects, following simple commands (e.g., pointing to body parts). But education may be a marker for something else that allows good recovery, such as discipline or determination (that led to a high education and may lead to greater participation in rehabilitation) or cognitive reserve. Alternatively, education may be a marker of economic resources (e.g., access to more rehabilitation) or may be correlated with healthy life style (lower rates of smoking, more exercise, and greater compliance with medications).

One previous study narrowed down the potential accounts of the effects of education. Education not only is associated with better recovery from aphasia but (to a lesser degree) is also associated with incidence and severity of impairment at onset in language tasks, even with 5th grade level of difficulty [40]. We studied 173 stroke patients within 24 hours of symptom development and hospitalized controls matched for age, education, and socioeconomic status (SES) with MRI and nine language tasks (auditory and written comprehension, naming (oral, written, and tactile), oral reading, oral spelling, written spelling, and repetition). Education was recorded in years, and SES was obtained from census tract data and assessed by mean neighborhood household income and family income. We found that the error rate for patients with 12th grade education or higher was significantly lower for auditory and written comprehension, written naming, oral reading, and spelling of fifth grade vocabulary words, even after adjusting for age, sex, stroke volume, and SES. These results indicate that even once learned, language performance may become less vulnerable to disruption by stroke with increasing years of education; and the effects of education cannot be explained by SES (a rough estimate of economic resources).

### 3.2. The Effects of Education on Recovery of Simple Attention to Space

In light of observed effect of education on language performance, we hypothesized that higher education might also be associated with better recovery of other focal cognitive functions. We evaluated the effect of education on hemispatial neglect, because it is a common but devastating impairment in spatial attention after right hemisphere stroke. We used a test that (1) can be performed easily by healthy children in first grade and (2) distinguishes between two forms of left hemispatial neglect failure to attend to the left side of the view (viewer-centered neglect) and failure to attend to the left side of individual objects on both sides of the view (stimulus-centered neglect), as shown in Figure 1.

To identify predictors of recovery of viewer-centered and stimulus-centered hemispatial neglect after acute right hemisphere stroke, we tested 35 patients with acute right hemisphere ischemic stroke at Day 1 and mean 32 weeks after stroke on the test shown in Figure 1, which distinguishes between viewer- and stimulus-centered neglect. They completed this task of detecting gaps in left or right sides of circles scattered across a page and MRI with PWI at Day 1 and then completed the same task at follow-up. Initial volumes of infarct and hypoperfusion were measured by a technician, who was masked to neglect scores. We identified variables associated with recovery of viewer-centered neglect (error rate in marking stimuli on left side of view) and stimulus-centered neglect (error rate in detecting left gaps in circles, irrespective of side of viewer) using multivariable regression. Age, education, volume of infarct, volume of hypoperfusion, initial error rate in marking stimuli on the left, initial error rate in detecting left-sided gaps in circles, and interval between onset and follow-up were entered as independent variables.

For the entire group of 35 patients, the average age was 58.4 years; and average education was 12.6 years. A total of 14 patients had viewer-centered neglect; 12 had stimulus-centered neglect; and 9 had both at onset. The degree of recovery in stimulus-centered neglect was associated with education and initial severity of stimulus-centered neglect (error rate in detecting left gaps at onset; *r* ^2^ = 0.59; *P* = 0.045). The degree of recovery in viewer-centered neglect was associated with education and initial severity of egocentric neglect; *r* ^2^ = 0.66; *P* < 0.0001, independently of other factors. Furthermore, the univariate (Pearson) correlation between education in years and accuracy in stimulus detection (egocentric neglect) was significant at follow-up (*r* = 0.57; *P* = 0.0003), but not at onset (*r* = 0.32; NS). These data show that the degree of recovery of both variants of neglect was positively correlated with higher education and lower initial severity of the specific type of neglect, independently of volume of infarct, volume of hypoperfusion, age, and time after onset of stroke [41].

Once again the effect of education on recovery may be somewhat surprising, particularly in this case, as the outcome we measured at follow-up does not require any formal education. Furthermore, better performance on the task at onset was not associated with higher education, while better* recovery* of performance was associated with higher education. These results indicate that people with higher education tend to show greater cognitive recovery after stroke. It will be important to identify the factors that mediate this association.

## 4. Conclusions

In this paper, we review a series of studies evaluating the consequences of stroke that have the greatest impact on quality of life and important variables that influence the degree of cognitive recovery after stroke. We unveil some surprising findings. First, while stroke treatment and outcome research to date has largely focused on recovery of very basic activities of daily living and motor function, survivors and their caregivers are more concerned with recovery of higher level cognitive functions, such as the ability to use written language and to empathize with others. Secondly, the degree to which an individual recovers even simple cognitive functions (which do not normally require formal education) is influenced by changes in blood flow in the early period and by their education level, as well as the size of their stroke or initial severity. That is, people with higher education make better recovery, although it is not yet clear whether this is a direct effect of education or whether a higher education is a marker for “cognitive reserve,” healthier lifestyle, or something else that might positively influence recovery. Independently of the positive effects of education, antidepressant use, particularly SSRIs, may also have positive effects on stroke recovery. Many of the studies reviewed here are relatively small, and so the findings need to be confirmed in large prospective studies and clinical trials, some of which are currently underway.

## Figures and Tables

**Table 1:** Sequelae reported by stroke survivors and their caregivers (in percent) who reported impairment as one of the “top 5” most important problems or moderate/important problems (*n* = 14 each group)^*^.

Domain^**^	Left hemisphere stroke survivor	Right hemisphere stroke survivor	Caregiver of left stroke survivor	Caregiver of right stroke survivor
Word retrieval	43	0	57	0
Reading	50	21	50	36
Writing/spelling	71	0	71	43
Memory	21	0	50	43
Energy (fatigue)	43	21	50	43
Mood	29	21	57	43
Walking	50	14	36	29
Right motor function	57	0	7	0
Left motor function	0	21	0	29
Prosody	0	0	0	29
Empathy	0	14	0	50
Spatial attention	0	0	0	29
Other cognitive	0	7	0	43
Personality/behavior	0	0	0	43
Sexual function	36	21	0	0

^*^Results from bilateral and brainstem stroke patients and their caregivers are not included as there were only 2 or 3 participants in each group.

^**^Other domains were not rated as moderate/important or in “top 5” most important problems by any participant (see the appendix for complete list of domains/symptoms).

## Appendix

### Items Included in the Questionnaire for Stroke Survivors and Caregivers

*Domain: Symptom(s) Rated by Survivor and Caregiver*

Right motor function:
  weakness in right arm;
  weakness in right leg;
  clumsiness/difficulty in using right hand;
  clumsiness/difficulty in using right leg.
Left motor function:
  weakness in left arm;
  weakness in left leg;
  clumsiness/difficulty in using left hand;
  clumsiness/difficulty in using left leg.
Walking/gait:
  difficulty in walking.
Swallowing:
  difficulty in swallowing.
Motor speech:
  difficulty in speaking.
Word retrieval:
  difficulty in word retrieval.
Auditory comprehension:
  difficulty in comprehending what other people say.
Spelling/writing:
  difficulty in spelling;
  difficulty in writing.
Reading:
  difficulty in reading.
Calculation:
  difficulty in calculating.
Music:
  difficulty in singing or in other aspects of music.
Empathy:
  difficulty in understanding the feelings of other people (loss of emotional empathy);
  difficulty in understanding the thoughts of other people (loss of cognitive empathy).
Prosody:
  difficulty in recognizing tone of voice and facial expressions in others;
  impaired use of tone of voice and facial expression to show emotion.
Spatial attention:
  difficulty in perceiving things on one side of space (“neglect”).
Attention:
  inattention.
Memory:
  impaired memory.
Other cognitive functions:
  other cognitive problems.
Other cognitive functions:
  other motor problems.
Vision:
  visual problems.
Extraocular movements:
  eye movement problems.
Mood:
  mood problems (circle one: depressed bipolar irritable manic other:__________).
Energy/fatigue:
  fatigue.
Sexual function:
  sexual dysfunction.
Personality/behavior:
  change in personality or behavior.
Sleep:
  impaired sleep.
Pain:
  pain (specify where:______________________________).
Sensation:
  sensory problems (specify where:________________________).
